# Amyloid Beta Peptide Is Released during Thrombosis in the Skin

**DOI:** 10.3390/ijms19061705

**Published:** 2018-06-08

**Authors:** Lilia Y. Kucheryavykh, Yuriy V. Kucheryavykh, A. Valance Washington, Mikhail Y. Inyushin

**Affiliations:** 1 Department of Biochemistry, School of Medicine, Universidad Central del Caribe, PO Box 60327, Bayamon, PR 00960-6032, USA; lilia.kucheryavykh@uccaribe.edu (L.Y.K.); yuriy.kucheryavykh@uccaribe.edu (Y.V.K.); 2 Department of Biology, University of Puerto Rico Rio Piedras, San Juan, PR 00936-8377, USA; anthony.washington@upr.edu; 3 Department of Physiology, School of Medicine, Universidad Central del Caribe, PO Box 60327, Bayamon, PR 00960-6032, USA

**Keywords:** natural peptide antibiotic, Aβ40 peptide, skin, thrombosis

## Abstract

While it is known that amyloid beta (Aβ) deposits are found in different tissues of both Alzheimer’s disease (AD) patients and healthy individuals, there remain questions about the physiological role of these deposits, the origin of the Aβ peptide, and the mechanisms of its localization to the tissues. Using immunostaining with specific antibodies, as well as enzyme-linked immunosorbent assay, this study demonstrated Aβ40 peptide accumulation in the skin during local experimental photothrombosis in mice. Specifically, Aβ peptide accumulation was concentrated near the dermal blood vessels in thrombotic skin. It was also studied whether the released peptide affects microorganisms. Application of Aβ40 (4 µM) to the external membrane of yeast cells significantly increased membrane conductance with no visible effect on mouse host cells. The results suggest that Aβ release in the skin is related to skin injury and thrombosis, and occurs along with clotting whenever skin is damaged. These results support the proposition that Aβ release during thrombosis serves as part of a natural defense against infection.

## 1. Introduction

In 1906, Dr. Alois Alzheimer showed that amyloid beta (Aβ) peptide is the major constituent of amyloid plaques in the brain, thus implicating these peptides in Alzheimer’s disease (AD) [1]. Aβ peptide and its β-sheet conglomerates were found not only in the brain, but also between muscle fibers associated with skeletal muscle myopathy [2,3] and in the eye [4,5,6]. In some cases of idiopathic cardiomyopathy, the presence of Aβ peptide in myocardium was also significantly increased in AD [7]. Aβ protein deposits in the skin were described many years ago, in and around the endothelium of dermal blood vessels in aged AD patients, and were proposed as a marker for this disease [8]. Later, skin accumulation of Aβ around blood vessels was found to be unrelated to the severity of symptoms in AD patients, occurring also in some healthy subjects [9]. Some authors also suggested a relationship between Aβ deposits in the skin and the occurrence of amyotrophic lateral sclerosis, as Aβ was detected in the skin of these patients at a higher level than in healthy individuals [10]. Two decades ago, Joachim et al. (1989) [8] pointed out that the source of Aβ deposits in the skin and other organs was probably a common blood-borne precursor. Recently, we showed that activated blood platelets aggregated in blood clots contribute to the accumulation of Aβ peptide in the brain: Aβ peptide was found in and around brain blood vessels in mice after thrombosis, as revealed by immunostaining, while in thrombocytopenic animals, this release was severely reduced [11].

Skin thrombosis is normally a defense against traumatic injury and/or a response to a number of other septic and aseptic causes that activate platelets in the skin and initiate the coagulation cascade. It is known that, besides coagulation factors, platelets also contain a relatively high concentration of amyloid precursor protein (APP), which mostly accumulates within α-granules, and full-length APP (containing Aβ peptide) is liberated upon platelet degranulation [12,13,14,15,16]. APP is itself a Kunitz-type protease inhibitor, which effectively inhibits chymotrypsin, trypsin, and other proteolytic enzymes [13,17,18], and promotes the activation of coagulation factor XII [19,20]. Therefore, platelet-released APP is likely to play an important role in the hemostasis and temporal stability of the thrombus [19].

Platelets are also the primary source (~90%) of Aβ peptide in human blood [21], while the Aβ peptide variants secreted by platelets are similar to those found in the senile plaques of AD patients [22]. When densely concentrated, platelets secrete mainly Aβ ending at residue 40 (Aβ40) as a final product, while the production of Aβ42 does not depend on platelet concentration [23]. Recently, it was shown that Aβ peptide has strong antibiotic activity against both Gram-negative and Gram-positive bacteria, as well as fungi and viruses [24,25,26,27]. Aβ peptide also protects mice against microbial infection in in vivo experiments [28]. Based on these findings, it was suggested that Aβ is a previously unrecognized antimicrobial agent that normally functions in the innate immune system [11,24,28,29]. However, Aβ peptide is not the only weapon in the platelet arsenal, as other antibacterial peptides were identified long ago [30,31,32,33,34]. Like Aβ, one of these antibacterial peptides (platelet microbicidal protein 1) has a variable length of either 72 or 73 amino acids, and is cleaved from a longer precursor [35].

While it was proposed that Aβ peptide oligomers aggregated into blood clot fibrils entrap microbes [28], we suggested another mechanism related to the plasma membrane pore-forming properties of Aβ peptide [11,29]. It was shown that soluble Aβ peptide oligomers at low concentrations perforate cell membranes by forming tetramer channels penetrable by K^+^ ions, and do so at higher concentrations by forming Ca^2+^-permeable hexameric pores [36,37,38]. An excess of Ca^2+^ permeability through these pores induces calcium dyshomeostasis, leading to cell death [39]. The same mechanism is employed by other natural peptide antibiotics with channel-forming activity, such as amphotericin B and nystatin [40,41].

We hypothesize that Aβ peptide (cleaved from the released APP or directly released by platelets) may form a sufficient number of large pores in the microbe plasma membrane to cause the death of the microorganism, thereby acting as part of the body’s defense system to prevent infection. This mechanism might be of special importance in the skin, which is the organ forming the initial defensive barrier.

Immunostaining and enzyme-linked immunosorbent assay (ELISA) methods were used in this study for in vivo evaluation of the release of Aβ in mouse skin during local experimental thrombosis. Patch-clamp electrophysiology methods were used to study the conductivity of the fungal cell membrane after the application of Aβ40 peptide. Mouse astrocytes were used as control eukaryotic host cells for the evaluation of the pore-forming activity of Aβ40 in eukaryotic cell plasma membranes.

## 2. Results

### 2.1. Aβ Immunoreactivity Was Concentrated in Dermal Blood Vessels a Few Minutes after Thrombosis Activation in the Skin and Became More Diffuse Later

**Figure 1:**
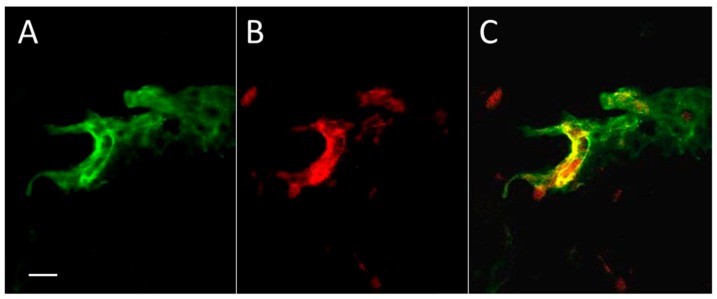
Aβ in the zone of experimental thrombosis in a mouse skin section. In addition, there is some diffuse anti-Aβ staining around the vessels. (**A**) The immunoreactivity against Aβ peptide (green); (**B**) The luminal surface of skin blood vessels marked by climbing shrub lectin (Rose Bengal, red); (**C**) Red + green = yellow color reveals the significant coincidence of Aβ immunoreactivity and blood vessel surface staining. Bar, 10 µm.

To study the distribution of Aβ peptide in skin after thrombosis, we applied a standard model of photo-induced coagulation to obtain thrombotic clots in skin blood vessels. Mice were injected intraperitoneally with the photoactivated dye Rose Bengal, and the bruised spot became visible after application of intense green laser light to the shaved skin. Immunocytochemical evaluation of coagulated and control skin samples revealed Aβ immunofluorescence in and around blood vessels (Figure 1), and mainly coinciding with the boundaries of the vessels. Some diffuse green staining of Aβ emanating from the vessels is also visible, as well as small parts of (apparently) other blood vessels.

**Figure 2:**
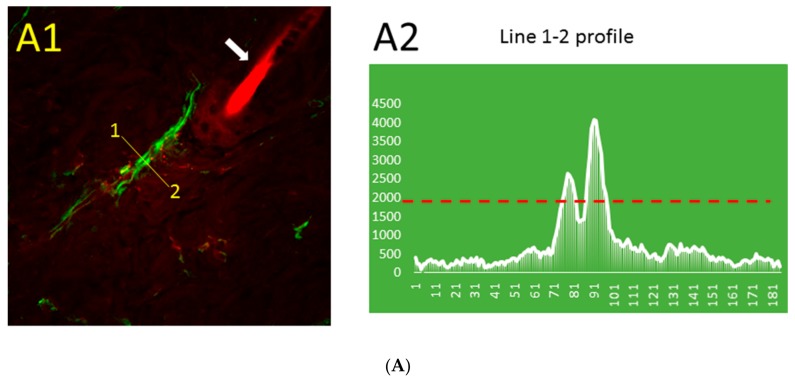

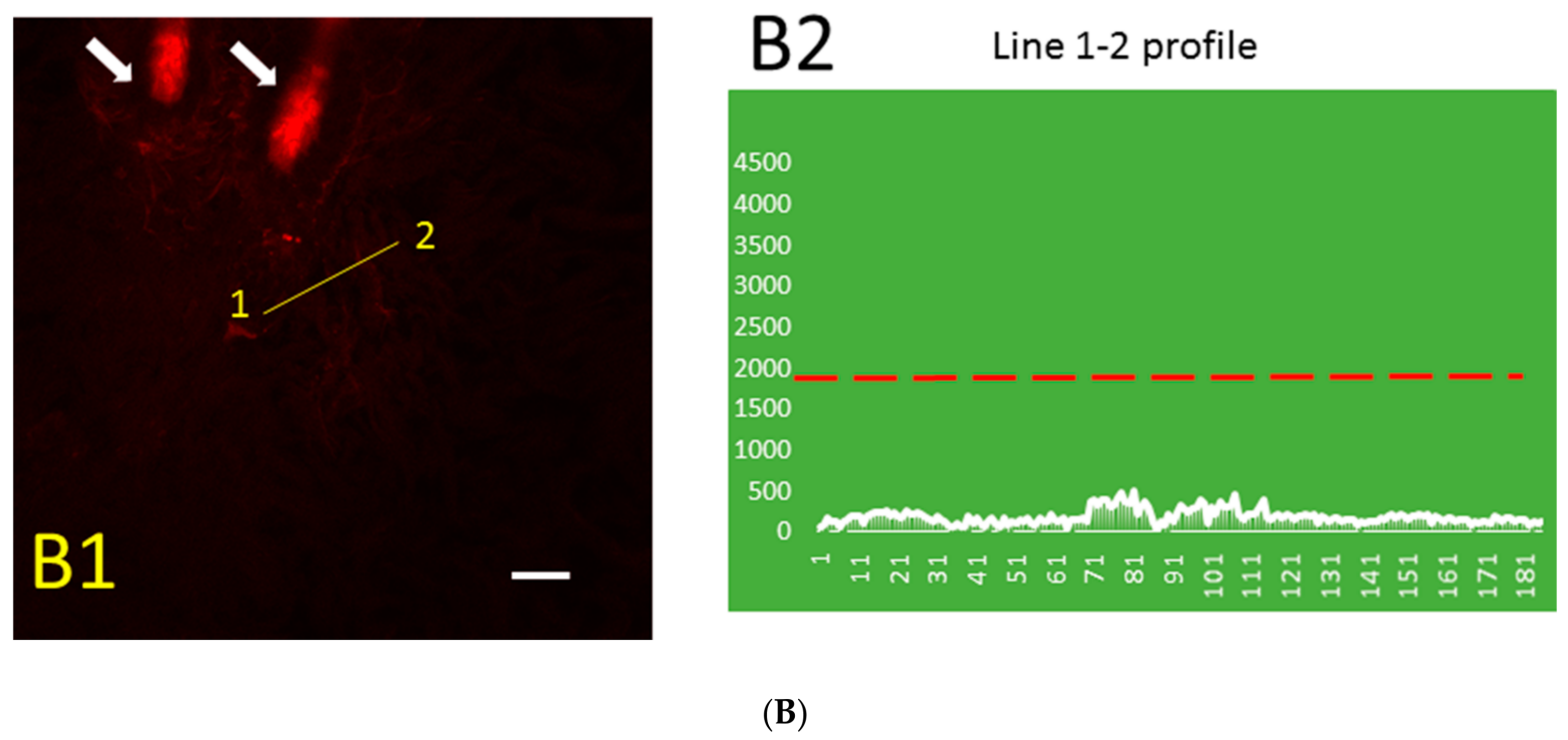
Distribution pattern of Aβ (green) immunofluorescence and blood vessel (red) fluorescence in thrombotic (**A1**) and control (**B1**) skin and the Aβ peptide profile (along line segment 1–2) in thrombotic (**A2**) and control (**B2**) skin in 0.5 µm steps. Green, immunofluorescence bound to Aβ peptide; red, Rose Bengal dye fluorescence bound to the luminal surface of blood vessels. Arrows, hair bulbs with visible hair shafts. The dashed red line in (**A2**) and (**B2**) indicates the intensity threshold of the green channel used in (**A1**) and (**B1**). Bar, 20 µm.

The distribution pattern of Aβ immunofluorescence in thrombotic (Figure 2A) and control (Figure 2B) skin was markedly different. The blood vessel stained for Aβ can be distinguished near the hair follicle (Figure 2A1), while similarly processed control skin has only a very low level of relatively uniform Aβ immunofluorescence that is not visible at the same threshold value (Figure 2B1). The line segment 1–2 (Figure 2A1) crosses the blood vessel, and the profile of green fluorescence along this line in Figure 2A2 shows Aβ diffusion from the blood vessel. The distribution of Aβ immunofluorescence along the randomly drawn 1–2 line segments in control skin (Figure 2B1) shows only a very low level of relatively uniform Aβ immunofluorescence (Figure 2B2).

The 3D distribution of Aβ, which is mainly concentrated within the large and small blood vessels, but to some extent leaks into the nearby tissue, is shown in Figure S1. Interestingly, erythrocytes were stained as Aβ-positive in a thrombotic skin sample (Figure S1). It is known that Aβ peptide in blood plasma binds to 98% of all erythrocytes in AD patients, and is a marker of the disease [42]. Moreover, the addition of synthetic Aβ not only marks erythrocyte membranes, but also makes them more elongated [43].

### 2.2. Formation of Blood Clots in the Skin Augments the Quantity of Aβ Peptide in Thrombotic Skin

**Figure 3:**
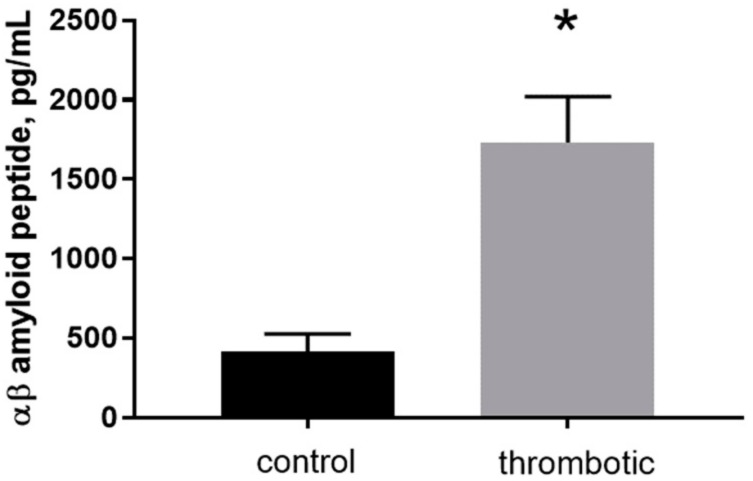
The concentration of free mouse Aβ40 peptide determined by enzyme-linked immunosorbent assay (ELISA) in control and thrombotic skin homogenate (pg/mL). Mean ± S.E. and significant differences between groups (*) are shown (*p* < 0.05).

To determine the levels of mouse-specific Aβ40 peptide in skin patches in control and post-photothrombosis skin a commercially available enzyme-linked immunosorbent assay (ELISA) was used. The final concentrations of Aβ40 in control (415 ± 57 pg/mL) and thrombotic (1733 ± 165.7 pg/mL) skin indicate an ~4-fold increase after thrombosis (Figure 3, *n* = 4, *p* < 0.001, *t* = 8.549, df = 5).

### 2.3. Application of Aβ40 to the External Membrane of Yeast Cells Visibly Augmented Membrane Conductance

**Figure 4:**
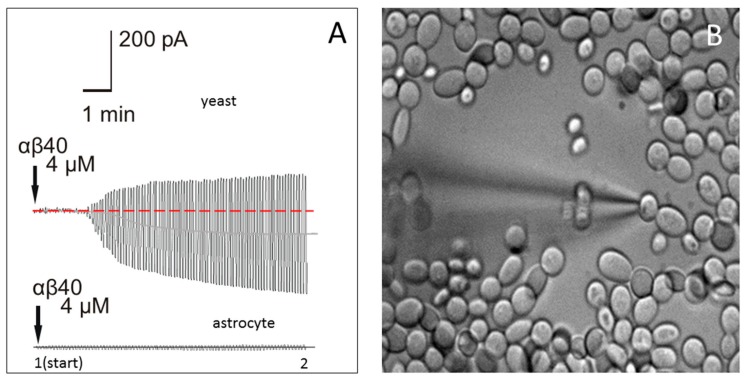
(**A**) Membrane currents in the cell-attached configuration in a yeast cell (upper trace) and a mouse astrocyte cell (lower trace) after Aβ40 peptide (4 µM) was added to the pipette solution and a test periodic voltage of ±100 mV was applied to the pipette; (**B**) Patch-clamp of yeast cells visualized with a difference interference contrast (DIC) microscope with 40× water immersion objective. Diameter of yeast cells, 3–4 microns.

Patch-clamp measurements with electrodes filled with synthetic Aβ40 were used in order to evaluate the effect of Aβ40 on membrane conductance in microorganisms. Yeast cells were selected as a model, because these microorganisms have the cell size and other characteristics that allow for the measurement of external membrane conductance using the patch-clamp technique (Figure 4B). Fine-tip electrodes (~10–12 MΩ tip resistance) were employed in the cell-attached voltage-clamp configuration without rupturing the cellular membrane. In this configuration, the cell is attached to the electrode with holding potential U, which is changed according to the protocol.

The current passing first through the electrode (R-elect = 10 MΩ), then (serially) through the patch of cellular membrane (R-patch), and then through the rest of the membrane (R-mem), is measured. A pore-former would affect only R-patch, while other resistances would stay constant, and an R-patch change would affect the total membrane current I (Figure 4A). Channel-forming peptides are not anchored, and rapidly diffuse around lipid membranes, forming temporary ion channels [44,45]. The resistance of the membrane patch perforated by a channel-former decreases, leading to a current increase. This resistance, and its changes with time, are calculated by measuring the current at the beginning (after attachment, time 1, Figure 4A) and after the effect is pronounced (time 2, Figure 4A), in which R = U/I, and the resistance change with time (Rtime1/Rtime2) can be calculated.

Four different concentrations of Aβ40 (0.4 nM, 40 nM, 400 nM, and 4 µM) were tested. While nanomolar concentrations of Aβ40 had no pronounced effect, 4 µM Aβ40 produced visible augmentation of the current amplitude from 20 pA to about 400 pA (Figure 4A, upper trace), which developed at 3–5 min after application. The effect was statistically significant, as 4 min after the electrode containing Aβ40 was attached to the yeast membrane, the resistance was reduced 17.4 ± 3.1-fold (*n* = 7, *p* < 0.0001, *t* = 245.7, df = 12). These changes in current indicate that the yeast membrane resistance was rapidly and significantly affected by the application of Aβ40.

### 2.4. Application of Aβ40 to the External Membrane of Mouse Astrocytes Did Not Significantly Affect Membrane Conductance

A standard patch-clamp was used in cell-attached configuration to study whether Aβ40 affects mouse cell membrane conductance at the same concentration as in yeast cells (4 µM). Astrocytes in a mouse hippocampus brain slice were used as the model. After an electrode containing 4 µM Aβ40 was attached to an astrocyte membrane (Figure 4A, lower trace), the transverse current in the astrocyte external membrane was not significantly affected 3–5 min after application (*n* = 8, *p* = 0.7584, *t* = 0.3136, df = 14). Thus, at a 4 µM concentration, Aβ40 had no immediate effect on the membrane resistance of astrocytes.

## 3. Discussion

This study established a correlation between the release of Aβ and thrombotic processes in mouse skin. Experimental photothrombosis was used, which is a well-established method for inducing rapid coagulation without mechanical damage to the tissue [11,46].

Immunostaining with a MOAB-2 (monoclonal mouse anti-Aβ antibody) confirmed that by 10 min after thrombosis, Aβ was present mainly in and around blood vessels in the skin (Figure 1, Figure 2 and Figure S1). The MOAB-2 antibody was chosen because of the high specificity to Aβ peptide. This antibody was extensively tested, previously, and found to be a pan-specific, high-titer antibody to Aβ residues 1–4, reacting with unaggregated, oligomeric, and fibrillar forms of Aβ42 and unaggregated Aβ40, as well as with aggregated amyloid in plaques [47,48]. The MOAB-2 anti-Aβ antibody did not detect APP or its derivatives, and did not colocalize with antibodies against either the N- or C-terminal of APP [47]. It is a technical challenge to stain the endothelial cells of blood vessels to study colocalization of Aβ and the blood vessel boundaries after thrombosis. The commonly used markers for blood vessel endothelium, CD31, von Willebrand factor (vWF), and CD34, are not suitable for staining endothelial cells in this model of platelet aggregation, because they are upregulated at the membranes of activated platelets in the clot, or are involved in platelet–vessel wall interactions, producing confusion [49,50,51]. For this reason, isolectin B4 (BSI-B4) from *Bandeiraea simplicifolia* was selected as the most suitable marker for the luminal surfaces of endothelial cells [52,53,54] in thrombotic models. Unlike thrombotic skin (Figure 2A1), in which the blood vessels were clearly marked with Aβ immunofluorescence, in control skin (Figure 2B1) there was no visible co-staining of Aβ and blood vessels or other structures. The Aβ immunofluorescence profile declined rapidly away from the blood vessel boundaries (Figure 2A2), while it stayed uniformly low in control skin (Figure 2B2).

Additionally, the study revealed that experimental thrombosis resulted in a 4-fold elevation of Aβ in skin, reaching 1733 ± 165.7 (pg/mL), which is about 0.4 nM. This concentration is in the range previously reported for Aβ in the cerebrospinal fluid (CSF) of young genetically modified 3xTg-AD mice, which spontaneously develop brain plaques [55]. The Aβ40 concentration in control skin in our experiments was 415 ± 57 (pg/mL), which is close to the range of concentrations (340–550 pg/mL) previously found in the blood plasma of healthy humans [56,57,58]. Measurement of Aβ in blood by ELISA reveals mainly free peptides, while a significant amount of Aβ peptide remains bonded to plasma proteins and lipoproteins [59], as well as to cell membranes [42], and our estimation of Aβ40 in skin has similar limitations. The detection of Aβ peptide generation in the skin is consistent with the hypothesis that a role of Aβ is as a natural defense against infection that accompanies thrombosis. The pore-forming properties of innate Aβ peptide might provide a rapid antimicrobial defense mechanism [11,29]. The current study provides additional support for this hypothesis, indicating that Aβ40 peptide significantly affects the conductivity of fungal membranes. Application of 4 µM Aβ40 to the external wall of yeast cells produced marked augmentation of the recorded current amplitudes (Figure 4A). Bertl et al. (1998) [60] first described the method for patch-clamping yeast cells without cell wall removal, showing that it forms a standard gigaseal. It is known that the fungal cell membrane is mechanically protected by a cell wall that is impossible to “open” by pressure application only [60,61]. Thus, the cell-attached configuration was used to record membrane currents, enabling this problem to be overcome. In yeast cells, a change in membrane resistance developed 2–5 min after peptide application, and became saturated after reaching a new limit. It should be mentioned also that the recommended Tris [tris(hydroxymethyl)aminomethane] buffer [60] was switched to HEPES [4-(2-hydroxyethyl)-1-piperazineethanesulfonic acid], because Tris buffer effectively blocks Aβ peptide-formed channels [62]. At the same time, the similar patch-clamp configuration and Aβ40 concentration had no effect on the resistance of the mouse astrocyte (host cell) external plasma membrane (Figure 4).

The pore-forming effect of Aβ peptide on brain cell membranes is well known [63,64]. The pronounced effect of Aβ40 in yeast (4 µM), but not in eukaryotic host cells in the current study, may reflect the fact that yeast cells have less cholesterol and more ergosterol in their membranes than mammalian cells [65], and it was previously found that membrane cholesterol reduces Aβ40 channel-forming activity [66].

Interestingly, there is a significant mismatch between Aβ40 peptide concentration found in the skin after thrombosis and its effective antimicrobial concentration. The results of this study demonstrate that, when Aβ40 is released in the skin during thrombosis, the concentration detected (~0.4 nM) is lower than necessary for a significant membrane effect in *Saccharomyces* yeast (4 µM). It was previously shown that pathologic *Candida* yeast are more sensitive to Aβ40 peptides (0.78 µg/mL, [24]); however, this was 1200-fold more than the concentration detected in the current study in thrombotic skin. At the same time, other antibacterial peptide species that were identified in platelets were found to be very effective against bacteria when applied in nanomolar concentrations as a thrombin-induced platelet supernatant, while purified proteins were effective in the 10–40 µM range [31,35]. Similarly, one might assume that the sensitivity of microorganisms to the native Aβ peptide would be significantly higher than the synthetic peptide.

Another explanation of this contradiction might be that the Aβ40 concentration that was determined by ELISA in mouse skin was not the local concentration around the damaged blood vessels, but rather the mean concentration in tissue. Aβ40 is mostly concentrated locally around blood vessels (Figure 2A,B), and rapidly declines away from the blood vessel boundary.

Many factors affect the sensitivity of microorganisms to Aβ40. First, the amount of oligomerized Aβ40 peptides depends on the solubilization method and the peptide form used in experiments. Oligomer formation by β-sheeting of monomeric Aβ is the necessary first step in forming Ca^2+^-permeable ion channels [67,68]. As an example, *Candida* yeast are very sensitive to Aβ peptides (0.78 µg/mL, [24]), but the recommended solubilization of the peptide with NH_4_OH [69] reportedly may lead to a lack of toxic effects at low concentrations [70], which supports the possibility that the solubilization method affects the oligomerization of Aβ.

Second, Aβ40 peptide carriers also reduce the effective concentration for pore formation. It was shown that in blood plasma, Aβ is usually attached to carriers, while unbound Aβ is rare. In blood plasma, Aβ40 was found bound to several proteins (that is, apolipoproteins A-I, A-IV, E and J; α2-macroglobulin; complement factors; immunoglobulins; transthyretin; apoferritin; and serum amyloid P component [71,72,73]), and one carrier is associated with many Aβ peptide molecules [74]. It is possible that if a carrier transports numerous Aβ40 peptide molecules to the cellular membrane and fuses to the membrane, it will drastically augment the local effective concentration of Aβ and result in pore formation. This carrier-based reduction of the bulk-phase Aβ40 concentration necessary for pore formation has been shown previously in artificial membranes [75]. When Aβ was concentrated inside liposomes, it was incorporated into plasma membranes more readily than by direct incorporation from aqueous solution.

We suggest that under natural conditions, blood plasma has liposome-like components, such as tissue-specific apolipoprotein type E (APOE), that facilitate the membrane-damaging action of amyloid-prone proteins and peptides. Under normal conditions, APOE participates in lipid transport and removes cholesterol and triglycerides from peripheral tissues. Many cell types produce APOE, including skin epithelium cells [76,77,78]. APOE was found in skin tissue, and plays an important role in primary localized cutaneous amyloidosis (PLCA) formation [79], which is associated with αβ deposits, as well as with deposits of misfolded amyloid-like keratin peptides [80]. Interestingly, the E4 allele of APOE is significantly increased in frequency in patients with PLCA [81], and similarly, the E4 allele is increased in frequency in AD [82]. Thus, APOE involvement in both types of amyloidosis makes it the main suspect as the amyloid peptide carrier and possible facilitator of the pore-forming effects of these peptides.

## 4. Materials and Methods

All procedures involving rodents were conducted in accordance with the National Institutes of Health regulations concerning the use and care of experimental animals. All procedures involving animals were approved by Universidad Central del Caribe Institutional Animal Care and Use Committee (Protocol #035-2017-16-01, 16 January 2017). All efforts were made to minimize suffering. For skin thrombosis experiments, animals were anesthetized with isoflurane (4% for induction, 1.75% for maintenance) using a Matrix Quantiflex VMC Anesthesia Machine for small animals (Midmark Corp., Dayton, OH, USA). After photothrombosis experiments, the anesthetized animals were rapidly decapitated and skin patches harvested. This euthanasia method is consistent with the recommendations of the Panel on Euthanasia of the American Veterinary Medical Association. In total, 12 C57BL/6 mice were used in these experiments.

### 4.1. Photothrombosis Model

In order to induce clot formation in mouse skin, a widely used method of photostimulated thrombosis was employed [11,46]. C57BL/6 mice of both sexes, 8–10 weeks old, were used in experiments. Briefly, prior to surgery, Rose Bengal (cat. #198250; Sigma-Aldrich Chemical Co., St. Louis, MO, USA) was dissolved in a sterile saline solution (7.5 mg/mL). The mice were then anesthetized with isoflurane, followed by peritoneal injection of Rose Bengal (150 µg/g animal weight), which was allowed to diffuse and enter the blood stream for 5 min. The animal skin was shaved and exposed to a laser beam (15 mW at 430 nm) until a visible bruise appeared on the skin (in ~10–15 min). The resulting thrombosis was 3–4 mm in diameter. The mice were killed, and control and thrombotic skin samples were harvested and fixed in 5% formaldehyde as soon after thrombosis formation (which takes ~10 min) as possible. Interestingly, alone among the many xanthene dyes, Rose Bengal exhibited a strong inhibitory effect on Aβ aggregation upon green photoexcitation, because of its high binding affinity for Aβ, and it also exhibits a remarkable red shift and a strong enhancement of fluorescence emission in the presence of Aβ [83]. Thus, Rose Bengal fluorescence is an indicator of the presence of Aβ.

### 4.2. Mouse Brain Slice Preparation

Mice (3 months old) were decapitated, and the brains were removed from the skull in ice-cold (2–4 °C) dissecting solution composed of (in mM): 126 NaCl; 2.5 KCl; 1.2 NaH_2_PO_4_; 7.0 MgCl_2_; 0.5 CaCl_2_; 25 glucose; 25 NaHCO_3_; and saturated with 95% O_2_ and 5% CO_2_. The hippocampal area from 4 animals were used for the brain slice preparations. Brain tissues were sectioned, and 250 μm slices were cut using a Vibroslicer (Leica VT 1000S, Leica Microsystems, Wetzlar, Germany). The slices were placed in an incubation chamber containing oxygenated artificial cerebrospinal fluid (ACSF; in mM: 126 NaCl; 2.5 KCl; 1.2 NaH_2_PO_4_; 1.0 MgCl_2_; 2.0 CaCl_2_; 25 glucose; 25 NaHCO_3_; and saturated with 95% O_2_ and 5% CO_2_) at 35 °C, and incubated at least 60 min before recording. Next, the slices were placed in the recording chamber (500 μL) and perfused with ACSF (35 °C) at a rate of 2 mL/min.

### 4.3. Preparation of Fungi (Yeast) for Patch-Clamping of Plasma Membrane

A common haploid *S. cerevisiae* yeast strain (BY4741) was used for patch-clamp experiments. Cells were grown overnight in a small volume of modified liquid YEPD medium (1% yeast extract, 2% peptone, and 5% d-glucose; cat. #Y1375; Sigma-Aldrich, St. Louis, MO, USA), with 30 mL per 125 mL Erlenmeyer flask at 25 °C with rotary shaking (90 rpm). The cells were harvested by centrifugation (500× *g* for 5 min) of 1 mL of suspension, and were then resuspended in the following buffer: 140 mM NaCl, 5 mM MgCl_2_, 10 mM CaCl_2_, 10 mM HEPES, adjusted to pH 7.5 with KOH. A few drops of this suspension were finally added to the patch-clamp chamber with the same buffer. Patch recording was done according to the method developed for *S. cerevisiae* by Bertl et al. (1998) [60] with modifications. The most important modification was the use of HEPES buffer instead of Tris, as Tris is a well-known blocker of Aβ channels [62]. Another modification was the use of the cell-attached patch-clamp procedure instead of whole-cell patch-clamp. Cells were visualized using an Olympus infrared microscope equipped with a difference of interference contrast (DIC) system (cat. #BX51WI; Olympus, Japan). Placed in the chamber, the cells sedimented rapidly, and under the microscope, cells (3–4 µm in diameter) were selected for patch-clamping. Borosilicate glass pipettes (O.D., 1.5 mm; I.D., 1.0 mm; World Precision Instruments, Sarasota, FL, USA) were pulled in four steps to a final resistance of 10–12 MΩ for recordings using a P-97 puller (Sutter Instrument Co., Novato, CA, USA). Electrodes were filled with the following solution: 175 mM KCI, 5 mM MgCl_2_, 4 mM ATP, 100 nM Ca^2+^, 1 mM EGTA, and brought to pH 7.0 with KOH. The necessary Aβ concentration was then added to this buffer. Currents were recorded in response to 2 s voltage steps, switching from +100 to −100 mV, with a 2 s holding interval at 0 mV between each pulse. Results are presented as concatenated response curves. Data were filtered at 1 kHz and sampled at 100 Hz during recording, and the presentation data were then filtered at 40 Hz. The pipette potential was not corrected.

### 4.4. Whole-Cell Recordings in Astrocytes

Membrane currents were measured using the single-electrode, whole-cell patch-clamp technique. Cells were visualized using an Olympus infrared microscope equipped with a DIC system (cat. #BX51WI; Olympus, Japan). A piezoelectric micromanipulator (MX7500 with MC-1000 drive; Siskiyou, Inc., Grants Pass, OR, USA) was used for voltage-clamp and current-clamp recording, and allowed the patch clamping of cells. A MultiClamp 700A patch-clamp amplifier with two separate patch-clamp channels paired to a DigiData 1322A interface (Molecular Devices, Inc., Sunnyvale, CA, USA) was used for recording and stimulation. The pClamp 10 software package (Molecular Devices, Inc.) was used for data acquisition and analysis. Borosilicate glass pipettes (O.D., 1.5 mm; I.D., 1.0 mm; World Precision Instruments; Sarasota, FL, USA) were pulled to a final resistance of 7–8 MΩ for recordings in four steps using a P-97 puller (Sutter Instrument Co., Novato, CA, USA). Electrodes were filled with the following solution (in mM): 130 K-gluconate, 10 Na-gluconate, 4 NaCl, 4 phosphocreatine, 0.3 GTP-Na_2_, 4 Mg-ATP, and 10 HEPES, and the pH was adjusted to 7.2 with KOH. Currents in the cell-attached configuration were recorded in response to 2 s voltage steps, switching from +20 to −20 mV, with a 2 s holding interval at 0 mV between each pulse. Data were filtered at 1 KHz and sampled at 100 Hz during recording. The presentation data were then filtered at 40 Hz, and the pipette potential was not corrected.

### 4.5. Preparation of Aβ Peptide Solution

We used lyophilized synthetic Aβ40 peptide trifluoroacetate salt (cat. #A4473; Sigma-Aldrich, St. Louis, MO, USA), and the 40 µM stock solution (~0.18 mg/mL) was prepared from the peptide on the day of experimentation by solubilizing Aβ40 in a buffer similar to electrode internal solution (see Section 2.2 and Section 2.3). Aβ stocks were sonicated for 5 min on ice, insoluble peptide aggregates were filtrated (0.2 µm pore filter), and the stock was then kept at +2 °C for storage. For experiments, the stocks were diluted into the same buffer that was used to fill the electrode, and once again, filtrated with a similar filter.

### 4.6. Immunohistochemistry and Confocal Microscopy

Immunostaining was performed using a protocol previously established in our laboratory [11]. Frozen 25 μm or 15 μm transverse sections from the skin photothrombosis area were generated. The sections were blocked with 5% normal goat serum/5% normal horse serum (Vector Laboratories, Burlingame, CA, USA) in PBS containing 0.3% Triton X-100 and 0.05% phenylhydrazine for 30 min, and then incubated with monoclonal mouse anti-Aβ antibody with very low cross-reactivity to APP (diluted 1:1000; cat. #LS-C181965, clone MOAB-2; LifeSpan Biosciences Inc., Seattle, WA, USA) in PBS-TAT (0.3% TritonX-100, 5% normal goat/5% normal horse serum, 1% sodium azide, 0.01% thimerosal) overnight at 4 °C. Fluorescein-5-isothiocyanate (FITC)-conjugated isolectin B4 (BSI-B4) from *Bandeiraea simplicifolia* (African climbing shrub; cat. #L2895; Sigma-Aldrich; St. Louis, MO, USA) was used to mark endothelial cells to study colocalization of Aβ with blood vessel boundaries. This isolectin is a known marker of the luminal surfaces of endothelial cells [52,53,54]. The sections were incubated with BSI-B4 (20 µg/mL), and the corresponding secondary antibodies (goat anti-mouse Alexa Fluor^®^ 546; Thermo Fisher Scientific, NY or fluorescein anti-mouse IgG; Vector Lab., Burlingame, CA, USA) overnight, rinsed twice in PBS, and visualized using an Olympus Fluoview FV1000 scanning inverted confocal microscope system equipped with a 60×/1.43 oil objective (Olympus, Melville, NY, USA). The images were analyzed using ImageJ software (http://imagej.nih.gov/ij) with the Open Microscopy Environment Bio-Formats library and plugin, allowing for the opening of Olympus files (http://www.openmicroscopy.org/site/support/bio-formats5.4/). The data were analyzed using custom colorization.

### 4.7. Enzyme-Linked Immunosorbent Assay (ELISA) Measurements

A specialized, ready-to-use, mouse-specific, solid-phase sandwich ELISA kit (cat. #KMB3481; Invitrogen) was used for direct measurement of the amount of Aβ40 peptide in the skin with and without thrombosis. The skin samples were homogenized mechanically, and 100 mg of homogenate was then lysed in guanidine solution (5 M guanidine HCl, 50 mM Tris HCl, pH 8.0). A monoclonal antibody to the NH_2_-terminus of mouse Aβ40 was coated onto the wells of the microtiter strips provided in the kit. Samples, including standards of known Aβ40 content for calibration purposes as well as experimental specimens, were pipetted into the wells. After washing, the rabbit antibody specific to the COOH-terminus of Aβ40 was added and detected with horseradish peroxidase-labelled anti-rabbit antibody. The optical density values at 450 nm were determined using a Wallac 1420 Victor2 Microplate Reader (PerkinElmer Inc., Waltham, MA, USA). A standard curve was used for final determination of the concentration of Aβ40 in the samples, and is presented as picograms of Aβ40 per milliliter of initial homogenate.

### 4.8. Statistics and Measurements

Using GraphPad Prism 7.03 (GraphPad Software, Inc., La Jolla, CA, USA) for calculations, an unpaired *t*-test was employed to estimate statistical differences. Values were determined to be significantly different if the two-tailed *p* value was <0.05.

## 5. Conclusions

The results of the study suggest that Aβ release in skin is associated with clot formation. Aβ affects membrane conductance in yeast microorganisms, but not in mouse somatic cells. Just as clotting is denoted as a “normal” protection and repair process in damaged skin, so does the liberation of Aβ during this process also lead to protection and repair. Skin-associated release of Aβ may also have implications for the development of treatment methods to cure AD, as patients with compromised clotting, as in thrombophilia, thrombophlebitis, or similar conditions, could have additional inflammation problems from an anti-Aβ vaccine. The development of the ACC-011 AD vaccine (Elan-Wyeth Corp., Dublin, Ireland) was halted due to a patient developing skin lesions, which was identified as a suspected case of inflammation of skin blood vessels [84]. Another implication of systemic generation of Aβ is the possible impact on the development of brain damage in AD. This aspect was directly confirmed recently in a study using constant transfusion of blood between genetically modified animals that developed Aβ plaques in the brain and their wild-type littermates. Aβ originated from transgenic AD model mice entered the circulation and accumulated in the brains of wild-type mice [85].

Generation of Aβ during thrombosis in the skin supports the hypothesis that Aβ release is a natural defense against infection that may accompany skin trauma. In support of an Aβ peptide role in natural antimicrobial defense, this study demonstrated that Aβ directly affects the fungal cell external membrane while not affecting the host cell membrane at the same concentration. This remarkable specificity for microbes, with relatively low toxicity for host cells, was shown previously for many antimicrobial peptides from marine invertebrates [86], and we suggest that there is some similarity to the Aβ40 effect.

## Supplementary Materials

The following are available online at http://www.mdpi.com/1422-0067/19/6/1705/s1.

## Abbreviations

Aβ	amyloid beta peptide
AD	Alzheimer’s disease
