# Supplementary material for: Amyloid Beta Peptide Is Released during Thrombosis in the Skin
Source: Int J Mol Sci. 2018 Jun 8;19(6):1705. doi: 10.3390/ijms19061705 (PMC6032379; doi:10.3390/ijms19061705)
Supplement: Supplementary file 1 [file ijms-19-01705-s001.zip › ijms-294593-supplementary.pptx]

## Slide 1
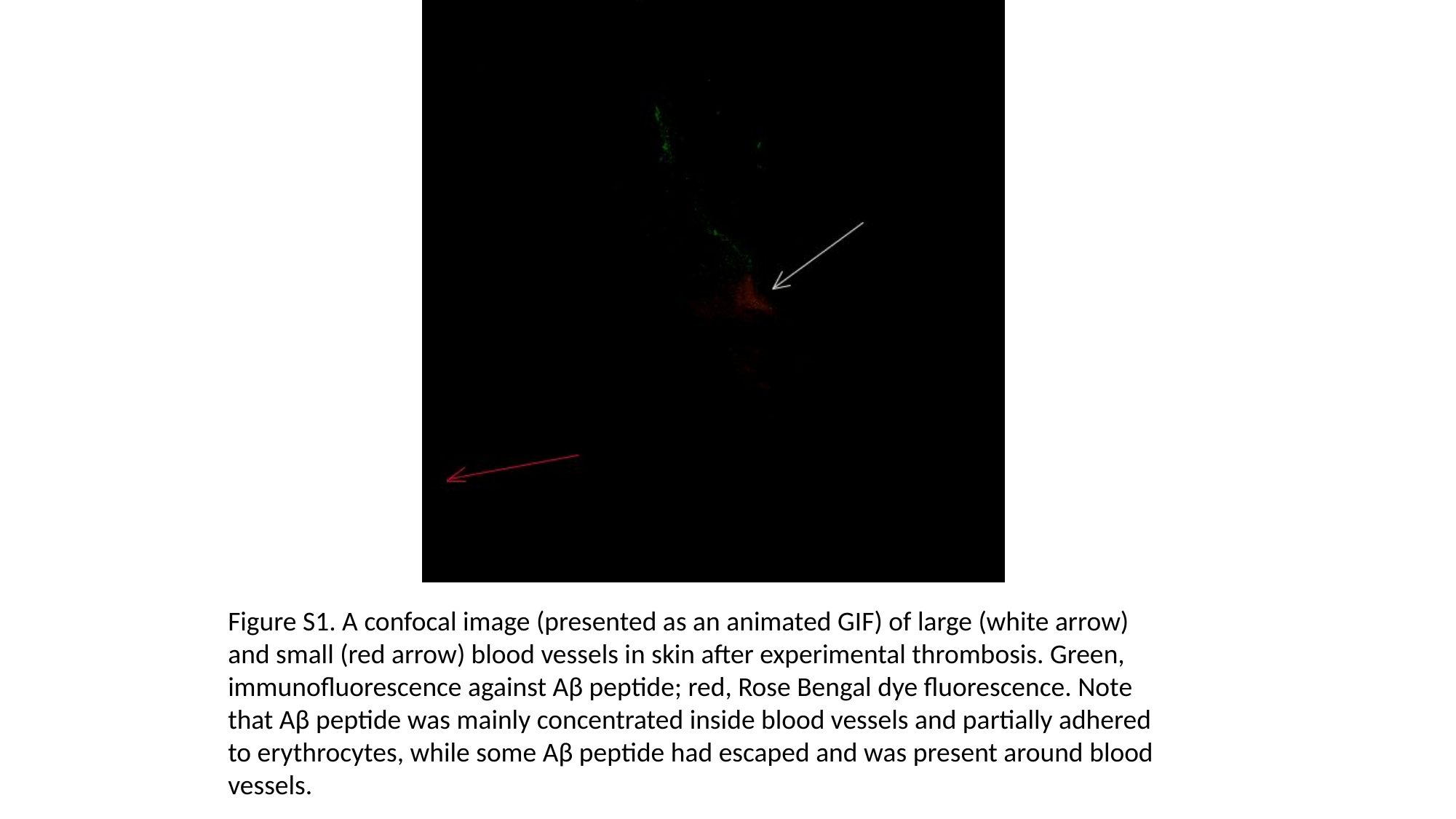

Figure S1. A confocal image (presented as an animated GIF) of large (white arrow) and small (red arrow) blood vessels in skin after experimental thrombosis. Green, immunofluorescence against Aβ peptide; red, Rose Bengal dye fluorescence. Note that Aβ peptide was mainly concentrated inside blood vessels and partially adhered to erythrocytes, while some Aβ peptide had escaped and was present around blood vessels.
